# Characterizing the bacteriophage PKp-V1 as a potential treatment for ESBL-producing hypervirulent K1 *Klebsiella pneumoniae* ST258 isolated from veterinary specimens

**DOI:** 10.14202/vetworld.2024.2008-2016

**Published:** 2024-09-08

**Authors:** Muhammad Usama Tariq, Saima Muzammil, Usman Ali Ashfaq, Muhammad Imran Arshad, Muhammad Shafique, Hasan Ejaz, Mohsin Khurshid, Lienda Bashier Eltayeb, Bi Bi Zainab Mazhari, Mohammed Yagoub Mohammed Elamir, Helal F. Al-Harthi, Muhammad Hidayat Rasool, Bilal Aslam

**Affiliations:** 1 Institute of Microbiology, Government College University Faisalabad, Faisalabad, Pakistan; 2 Department of Bioinformatics and Biotechnology, Government College University Faisalabad, Faisalabad, Pakistan; 3 Institute of Microbiology, University of Agriculture, Faisalabad, Pakistan; 4 Department of Clinical Laboratory Sciences, College of Applied Medical Sciences, Jouf University, Sakaka 72388, Saudi Arabia; 5 Department of Medical Laboratory Sciences, College of Applied Medical Sciences, Prince Sattam Bin Abdulaziz University-Al-Kharj, 11942 Riyadh, Saudi Arabia; 6 Department of Clinical Laboratory Sciences, College of Applied Medical Sciences, Jouf University, Qurayyat 75911, Saudi Arabia; 7 Biology Department, Turabah University College, Taif University 21995, Saudi Arabia

**Keywords:** antibiotic resistance, bacteriophage, *Klebsiella pneumoniae*, veterinary

## Abstract

**Background and Aim:**

The dearth of new antibiotics necessitates alternative approaches for managing infections caused by resistant superbugs. This study aimed to evaluate the lytic potential of the purified bacteriophage PKp-V1 against extended-spectrum β-lactamase (ESBL) harboring hypervirulent *Klebsiella pneumoniae* (hvKp)-K1 recovered from veterinary specimens.

**Materials and Methods:**

A total of 50 samples were collected from various veterinary specimens to isolate *K. pneumoniae*, followed by antimicrobial susceptibility testing and molecular detection of various virulence and ESBL genes. Multilocus sequence typing of the isolates was performed to identify prevalent sequence types. The bacteriophages were isolated using the double-agar overlay method and characterized using transmission electron microscopy, spot tests, plaque assays, stability tests, and one-step growth curve assays.

**Results:**

Among 17 (34%) confirmed *K. pneumoniae* isolates, 6 (35%) were hvKp, whereas 13 (76%) isolates belonging to the K1 type were positive for the wzy (K1) virulence gene. All (100%) hvKp isolates exhibited the allelic profile of ST258. Overall, PKp-V1 exhibited an 88 % (15/17; (p ≤ 0.05) host range, among which all (100 %; p ≤ 0.01) hvKp isolates were susceptible to PKp-V1. PKp-V1 exhibited a lytic phage titer of 2.4 × 10^8^ plaque forming unit (PFU)/mL at temperatures ranging from 25°C to 37°C. The lytic phage titers of PKp-V1 at pH = 8 and 0.5% chloroform were 2.1 × 10^8^ PFU/mL and 7.2 × 10^9^ PFU/mL, respectively.

**Conclusion:**

Although the incidence of ESBL-infected *K. pneumoniae* in veterinary settings is worrisome, PKp-V1 phages showed considerable lytic action against the host bacterium, indicating the potential of PKp-V1 as a possible alternative therapeutic option against MDR *K. pneumoniae*.

## Introduction

During the last decade, *Klebsiella pneumoniae* has emerged as a substantial health concern because of the increasing incidence of infections worldwide [1]. According to the World Health Organization, *K. pneumoniae* is a critical public health threat [2]. The ubiquitous dissemination of this pathogen is well reported; animals such as cows, horses, and many other domestic animals are common hosts of *K. pneumoniae* [3–5]. The incidence of *K. pneumoniae* in the dairy cow gut is higher than that in the human gut [6]. Interestingly, other than wild and domestic animals, *K. pneumoniae* has also been isolated from bird feces, insects, and fish [7, 8]. It has been proposed that the transmission of *K. pneumoniae* into the human gut is largely associated with animal-derived food products, which, in turn, increases the overall burden of the pathogen in the natural environment [4]. Common infections caused by *K. pneumoniae* in domestic animals include respiratory tract infections, blood infections, cervix inflammation, and epidemic metritis [9]. Animal-associated Mastitis and pneumoniae are also common clinical manifestations of *K. pneumoniae* [10]. Consequently, such infections may lead to considerable production losses and economic impacts on the dairy industry [2].

The control of multidrug-resistant (MDR) *K. pneumoniae* is challenging because of the limited treatment options resulting from the scarcity of novel antibiotics. Consequently, alternative therapeutic approaches are necessary to manage the spread of this lethal pathogen. Phage therapy, a promising alternative, was developed to treat bacterial infections even before the discovery of the first antibiotic, penicillin. Bacteriophages, or phages, are viruses that specifically target bacterial hosts and play a crucial role in balancing bacterial populations in the environment [11, 12]. The advantages of using phages over antibiotics include their host specificity, ability to degrade biofilms, and low toxicity to humans and animals [13]. Furthermore, phages are considered self-replicating drugs because they precisely target and eradicate specific bacterial populations when localized at the site of infection [14].

*K. pneumoniae* is a pressing global health threat with significant economic implications. The financial burden of treatment and production losses is particularly concerning for developing countries like Pakistan. Recently, we evaluated the lytic activity of bacteriophages against clinical isolates of *K. pneumoniae* and found that the isolated phages exhibited promising lytic potential. This suggests that phages could be considered an alternative approach to combating the antimicrobial resistance crisis [15, 16]. In this study, we investigated the lytic potential of PKp-V1 against extended-spectrum β-lactamase (ESBL)-producing hypervirulent *K. pneumoniae* K1 (hvKp-K1) isolated from veterinary specimens.

## Materials and Methods

### Ethics approval and informed consent

Ethical approval for this study was obtained from the Ethical Review Board of Government College, Faisalabad, Pakistan (GCUF/ERB/21/67). Informed consent was obtained from the owners/administrators of the farms for sample collection. The collection of specimens complied with institutional board guidelines.

### Study period and location

The study was conducted during September 2022 to June 2023 at the Institute of Microbiology, Government College University, Faisalabad, Punjab, Pakistan.

### Sample collection and processing

A total of 50 samples were collected using a convenient sampling technique [17]. Samples were collected aseptically from various specimens, including cattle/buffalo pus samples (n = 14), sheep/goat oral swabs (n = 17), fecal samples (n = 9), and samples from infected teats (n = 10). To isolate *K. pneumoniae*, all samples were first streaked onto nutrient agar, followed by MacConkey agar plates. For further purification of the isolates, a HiChrom selective agar base (HiChrom™ Klebsiella selective agar M1573-500G; HiMedia Laboratories Pvt. Ltd., Mumbai, India) was used [18–20]. Conventional microbiological methods were adopted for the identification of isolates, for example, API 20E kits (BioMérieux, France) [16, 21].

### Phenotypic characterization of hvKp

The test is considered positive if a vicious string of more than 5 mm in length is formed when a sterilized loop is touched to a colony of *K. pneumoniae* on selective agar and stretched from that point. This test was performed to assess the hypermucoviscosity phenotype, which is an established virulence factor of hvKp [22]. The extended thread (>5 mm) of the *K. pneumoniae* colony was considered hvKp.

### Detection of virulence genes

Genotypic detection of hypervirulent isolates was performed through Polymerase Chain Reaction (PCR) by amplifying several genes encoding virulence factors, including *wzy/magA* (K1; mucoviscosity associated gene)*, rmpA* (regulator of phenotype A), *fimH* (fimbriae type I), and *ybtA* (gene of iron acquisition system). The primer details are described in Table-1. The PCR amplicons were subjected to 1.2% agarose (ThermoFisher Scientific®, USA) gel electrophoresis in 1X TBE buffer (40 mM Tris-HCl, 1.2 mL boric acid, 2.0 mM Ethylenediaminetetraacetic acid (EDTA), pH = 8.0), and the obtained bands were photographed using a gel documentation system (Bio-Rad®, USA).

**Table-1:** Sample-wise distribution of *K. pneumoniae* (host bacterium) isolates and their respective phage susceptibilities, along with statistical analysis.

Sample specimens		Total number of collected samples			No. of isolates (%)			MDR of positive isolates (%)		hvKp of positive isolates (%)			Phage susceptible isolates (%)	
Pus samples		14			6 (42)			1 (16)		1 (16)			6 (100)	
Oral swabs		17			5 (29)			1 (20)		2 (40)			4 (80)	
Fecal samples		9			3 (33)			3 (100)		2 (66)			3 (100)	
Infected teats		10			3 (30)			2 (66)		1 (33)			2 (66)	
Total		50			17 (34)			7 (41)		6 (35)			15 (88)	

**Statistical analysis**														

**PC**	**df**		**t stat**	**t alpha half, 95% CI**		**MD**	**SD**		**SE**		**Lower CL**	**Upper CL**		**T-test** **p-value**

0.943	3		1.732	3.182		0.5	0.577		0.288		−0.418	1.418		0.051

PC=Pearson correlation, MD=Mean difference, SD=Standard deviation, SE=standard error, CL=Confidence level

### Antibiotic susceptibility profiling of the isolates

The antibiotic susceptibility of the isolates was studied against different antibiotics using the modified Kirby-Bauer Disk Diffusion method. Briefly, a 0.5 McFarland suspension with 1 × 10^8^ colony forming unit (CFU)/mL was achieved by dissolving the bacterial growth in sterile phosphate buffer saline (PBS; 0.9%). The estimation was performed using a spectrophotometer (Hitachi-UH5300-UV-Vis/NIR, Hitachi High-Tech Corporation, Tokyo, Japan) as instructed. The antibiotic disks were fixed gently over Muller-Hinton (MH) agar (Oxoid, UK) plates having a bacterial lawn and were then incubated at 37°C overnight. The isolates (NCBI GenBank no. MF953599 and MF953600) of *K. pneumoniae* were used as positive controls. The specific inhibition zone for each antibiotic was estimated according to the Clinical and Laboratory Standards Institute 2020 criteria [23]. Subsequently, the broth microdilution method was used to estimate the minimum inhibitory concentration of the studied antibiotics [24].

### Detection of ESBL genes

For this purpose, DNA extraction was performed from the fresh culture of *K. pneumoniae* and quantified using NanoDrop™ (ThermoFisher Scientific). PCR amplification of different ARGs was performed using specific primers. Briefly, a total of 25 μL PCR mix was prepared to have 5 μL DNA template, 2 μL of F and R primers (100 pM), 8 μL DreamTaq (Thermo-Scientific™, USA), 10 μL SuperQ water (Ambion-AM9932; ThermoFisher Scientific). The specific annealing temperature was adjusted for each ESBL gene (details are given in Table-1. Afterward, the PCR amplicons were subjected to 1.2% agarose (ThermoFisher Scientific) gel electrophoresis in 1X TBE buffer (40mM Tris–HCl, 1.2mL boric acid, 2.0 mM EDTA, pH = 8.0), and acquired bands were studied using a gel documentation system (Bio-Rad).

### Multi locus sequence typing (MLST)

To observe the sequence type (ST) of the isolated *K. pneumoniae*, MLST was performed according to the MLST-Pasteur-scheme (https://bigsdb.pasteur.fr/klebsiella/klebsiella. html). For this purpose, the previously prescribed method was used [25]. Briefly, PCR (Bio-Rad) was performed to amplify seven housekeeping genes of *K. pneumoniae*: *rpoB, gapA, infB, phoE*, *tonB, mdh*, and *pgi*. Oligos were prepared by Integrated DNA Technologies Inc. (California, USA). PCR for MLST was performed with specific conditions: initial denaturation for 3 min at 94°C, followed by 35 cycles with 30 s of denaturation, 30 s of annealing at 50°C (expected tonB 46°C, gapA 59°C, 30 s of extension at 72°C, and at the end 5 min of final extension at 72°C. Subsequently, 1.5 % agarose (CSL-AG500; Cleaver Scientific, UK) gel electrophoresis was performed to determine the PCR amplicons.

### Sample collection for bacteriophage isolation

For bacteriophage isolation, veterinary sewerage samples (n = 50) were taken from various veterinary healthcare facilities and abattoirs. For this purpose, 500 mL of sewerage water was placed in disposable plastic containers. The collected samples were processed for bacteriophage isolation and enrichment [26].

### Phage enrichment and purification

The samples were initially treated with 1% chloroform and then subjected to phage enrichment after 2 h. In total, 10 mL of 10X L.B. broth (Oxoid, UK) was decanted to cover flasks. A water sample (90 mL) was added to L.B broth containing flasks. Afterward, 100 μL of freshly grown *K. pneumoniae* was inoculated into flasks and incubated in a shaking incubator at 70 rpm at 37°C overnight. Subsequently, flasks were centrifuged at 18000 × *g* for 15 min and subjected to double-agar assay as previously described by Zhang *et al*. [27]. A single plaque was collected for further phage purification [28].

### Spot test

The host ranges of isolated phages were studied using spot assays. In summary, 100 μL of *K. pneumoniae* culture was poured over LB agar plates; then, 10 μL of diluted phage lysate was poured over and incubated overnight at 37°C. The previously described procedure was adopted for the routine test dilution (RTD), that is, the first dilution of the phage that formed clear and enumerable plaques on a propagated bacterial lawn [29]. A total of 100× RTD of the tested phages were detected for the *K. pneumoniae* isolates, followed by overnight incubation at 37°C. Overall, 50% of plaque formation on propagated *K. pneumoniae* growth lawns was considered strong positive RTD.

### Morphological characterization by transmission electron microscopy (TEM)

The studied phages were subjected to TEM for morphological characterization. The National Institute of Biotechnology and Genetic Engineering, Punjab, Pakistan, provided TEM analysis services. Briefly, the phage suspension was fixed over a formvar-carbon-coated grid Cu Mesh 300 with 1% glutaraldehyde, followed by 2% uranyl acetate negative staining and detailed examination under an EM microscope (Zeiss™, Germany) at 100 kV.

### Stability of the isolated phage in different conditions

The stability of the isolated bacteriophage was tested at different pH, temperature, and chloroform levels, as previously described by Aslam *et al*. [16]. Thermal stability was observed at temperature ranges from 0°C to 55°C. In total (100 μL) 10^6^ PFU/mL of phage lysate were incubated for approximately 4 h at the studied temperature range. Phage titers were then estimated using the double-agar overlay method [30]. Similarly, the isolated phages were tested at pH 2, 4, 7, 9, and 11. Various concentrations (1%, 1. 5%, and 2%) of chloroform were used for bacteriophage stability testing. In total (100 μL) 10^8^ PFU/mL of phage lysate was dispensed in PBS (900 μL) along with different chloroform concentrations individually [31].

### Phage adsorption assay (PA)

PA was performed to estimate the adsorption rate of the isolated phages. Fresh MH broth-grown *K. pneumoniae* (10^8^ CFU/mL) was incubated with an isolated phage (10^6^ PFU/mL) suspension. Downstream, centrifugation was performed for 10 min at 12,000 × *g*, followed by filtration (0.22 μm). Free phages were estimated using double-layer agar assays because the reduced phage titer was considered phage-host adsorption.

### One-step growth

A one-step growth assay was performed as described by Ahmad *et al*. [32] to study the infection cycle of isolated phages. In the test adsorption period, the latent phase and the burst size were determined. Briefly, 0.9 mL of fresh *K. pneumoniae* culture (10^8^ CFU/mL) and 0.1 mL of phage multiplicity of infection (MOI = 0.01) lysate were mixed and incubated at 37°C for 5 min. This blend was diluted, and 0.1 mL samples were taken up to 30 min at 5-min intervals, followed by up to 90 min at 15-min intervals. Again, a double agar overlay assay was performed to count phage plaques.

### Biofilm inhibition and eradication assay

The anti-biofilm activity of the isolated phage was determined against the host bacterium K. *pneumoniae* as previously described by Aslam *et al*. [16]. Briefly, bacterial growth (OD_600_ = 0.5) was diluted in L.B broth at 10:100 ratios and dispensed into the first column of a microtitration plate. A 100 μL phage suspension with a 0.01 MOI was added to all column wells containing the host bacterium. Subsequently, incubation was performed at 37°C for 10 h, and planktonic cells were discarded. Anti-biofilm activity was observed on mature biofilm produced by the host bacterium after inoculating 100-μL a phage (10^8^ PFU/mL) diluted with water. Washing of the columns was performed, and the plates were air-dried. Afterward, 25 μL crystal violet (2%) solution was dispensed in all wells and incubated for 20 min. After washing with ethanol (150 μL), the bacterial broth culture in the second column was used as a positive control. An ELISA plate reader (Multiskan™ FC Microplate Photometer Thermo Scientific) set to optical density (OD_600_) was used to study the reduction in OD.

## Results

### Isolation of hvKp-K1 ST258

Out of 50 samples, 17 (34%) were identified as *K. pneumoniae*. Of these confirmed *K. pneumoniae* isolates, 7 (41%) were MDR isolates, whereas 6 (35%) isolates were recorded as hvKp. Overall, the incidence of *K. pneumoniae* among the various sample specimens was 42%, oral swabs (29%), fecal samples (33%), and infected teats (30%). The distributions of isolates from various specimens are presented in Table-1.

After phenotypic detection, PCR was performed to detect virulence genes, and the results revealed that most of the isolates belonged to the K1 serotype. A total of 13/17 (76%) isolates were positive for *way* (K1) virulence, whereas other virulence genes like *fimH*, *rmpA*, and *ybtA* were found in 14/17 (82%), 4/17(23%), and 1/17 (6%) of the isolates, respectively (Table-1).

Overall, three different STs were observed in the study, namely, ST29 (7/17; 41%), ST258 (6/17; 35%), and ST11 (4/17; 23%). However, all (6/6; 100%) hvKp exhibited the allelic profile of ST258, which was also used as a host bacterium for the evolution of the lytic activity of the isolated phage.

### Antibiotic susceptibility profile

**Figure-1:**
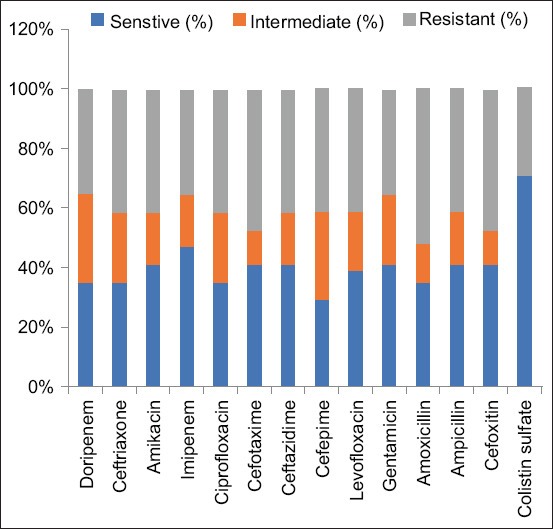
Antimicrobial susceptibility profiles of the isolates.

Overall, out of the confirmed isolates, 7 (41%) were detected as MDR *K. pneumoniae*. A total of 3 (17%) isolates were observed as MDR-hvKp. Resistance to various antimicrobial agents ranges from 29% to 52% (Figure-1).

Genotypic detection of ARGs showed that the distribution of ESBL genes among MDR isolates *of K. pneumoniae* were *bla*SHV (6/7; 86%), *bla*TEM (6/7; 86%), *bla*CTX-M (5/7; 71%), *bla*OXA-48 (4/7; 57%), and *bla*NDM-1 (1/7; 14%) (Table-2).

**Table-2:** Primer details and gene distribution among *K. pneumoniae* isolates.

Gene name	Primers sequence	Annealing temperature (°C)	Percentage prevalence
*Wzy* (K1)	F-GGTGCTCTTTACATCATTGC R-GCAATGGCCATTTGCGTTAG	47	13/17 (76)
*rmpA*	F-ACTGGGCTACCTCTGCTTCA R-CTTGCATGAGCCATCTTTCA	48	4/17 (23)
*fimH*	F: GCTCTGGCCGATACCTACCGACGG R: GCGAATAGATAACGTCGCCTGACGG	48	14/17 (82)
*ybtA*	F: ATGACGGAGTCACCGCAAAC R: TTACATCACGCGTTTAAAGG	49	1/17 (6)
*bla*_TEM_	F-TCAACATTTCCGTGTCG R-CTGACAGTTACCAATGCTTA	42	6/7 (86)
*bla*_SHV_	F-ATGCGTTATATTCGCCTGTG R-AGATAAATCACCACAATGCGC	47	6/7 (86)
*bla*_CTX-M_	F- GGATATCGTTGGTGGTGCCATA R-TTTGCGATGTGCAGTACCAGTAA	60	5/7 (71)
*bla*_NDM-1_	F-TGCCCAATATTATGCACCCGG R-CGAAACCCGGCATGTCGAGA	60	1/7 (14)
*bla*_OXA-48_	F-TTGGTGGCATCGATTATCGG R-GAGCACTTCTTTTGTGATGGC	56	4/7 (57)

### Isolation of lytic bacteriophages from MDR *K. pneumoniae*

**Figure-2:**
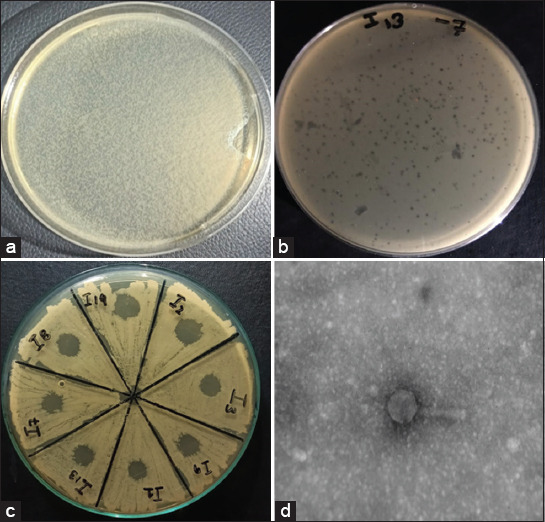
(a) Plaque assay using double-agar overlay for PKp-V1 purification, (b) Detection of PKp-V1 using the spot test after initial enrichment, (c) Determination of *the* PKp-V1 host range using the spot test, and (d) TEM analysis of isolated bacteriophages.

Overall, among the total collected samples, 8 (16%) were positive for lytic phages of the host bacterium *K. pneumoniae* (Figures-2a and b). The isolated PKp-V1 bacteriophage with a significant host range was selected for further characterization.

### Host range of PKp-V1

The results were observed as clear zones of spots due to the lytic activity of PKp-V1. Overall, PKp-V1 showed a host range of 88 %, as it showed significant (p ≤ 0.05) lytic activity against the isolates (Figure-2c). Moreover, PKp-V1 showed lytic activity against 6 (85%) MDR isolates, whereas all 3 (100%; p < 0.01) MDR hvKp were susceptible to PKp-V1 lytic activity (Figure-2). The average plaque size and formation time was estimated to be +3 mm and +9 h, respectively.

Morphology of the phage studied through TEM revealed that the isolated phage, that is, PKp-V1 has an icosahedral head with a neck associated with a long tail and appropriately belongs to the order of tail phages known as *Caudovirales*. For detailed visualization, head diameter and tail length were observed, and PKp-V1 exhibited the morphological characteristics of the *Myoviridae* family (Figure-2d).

### Stability testing of PKp-V1

**Figure-3:**
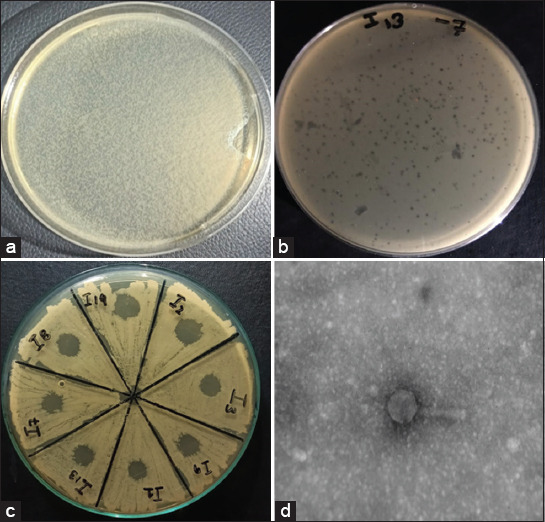
(a) Comparative analysis of PKp-V1 titers at different temperatures, (b) Comparative analysis of PKp-V1 titers at various pH, (c) Comparative analysis of PKp-V1 titers at various chloroform concentrations, and (d) One-step growth analysis of PKp-V1 infection.

During stability testing, we observed that temperature had a considerable impact on phage growth and plaque formation. Although satisfactory lytic activity was observed at 25°C as well, the optimum temperature for the lytic activity of PKp-V1 against *K. pneumoniae* was 37°C ± 1 with a phage titer of 2.4 × 10^8^ PFU/mL, which indicates that they belong to a high temperature-phage. No plaque formation was observed at 0°C and 43°C (Figure-3a).

In the case of pH, plaques were not seen at pH=3, while a few plaques were detected at pH=5. The significant plaque titer was calculated at pH=7 and pH=8, that is, 2.1 × 10^8^ PFU/mL. Similarly, no plaques were observed at high pH, that is, 11 (Figure-3b). For chloroform, after 5–6 h of incubation at concentrations ranging from 0.5% to 1.5% chloroform, PKp-V1 was found to be stable, and plaques were observed against the host bacterium, that is, *K. pneumoniae* (Figure-3c).

### One-step growth analysis

The analysis was performed to determine the infection activity of PKp-V1 in terms of the adsorption time, latent period, and burst size. PKp*-*V1 displayed an adsorption time of 9 ± 1 min, whereas the increase in phage titer showed that the latent period of PKp-V1 was 27 ± 3 min, covering a 9-min period of adsorption. PFU/cell is known as the burst size and is calculated as the ratio of PFU/mL at the plateau to PFU/mL at the first rise. PKp-V1 exhibited a burst size of 197 PFU/cell for the host bacterium (Figure-3d).

### Biofilm inhibition assay

Reductions in OD were observed and considered the anti-biofilm ability of the isolated PKp-V1 phage. Overall, OD_600_ mean of mature biofilm produced by *K. pneumoniae* was 0.392. The mean post-phage treatment mean (OD_600_) was 0.107. A significant reduction in OD_600_ was observed, which suggested the strong anti-biofilm ability of PKp-V1.

## Discussion

*Klebsiella pneumoniae* is a veterinary pathogen in various settings around the world [33]. This study aimed to evaluate the lytic potential of the isolates phages against hvKp isolated from veterinary specimens. Given the limited treatment options available for MDR *K. pneumoniae*, we also investigated the lytic potential of PKp-V1. Our findings suggest that PKp-V1 is a promising alternative therapy against this deadly pathogen.

hvKp was significantly distributed among various veterinary specimens, highlighting the hygienic conditions of local veterinary settings. Previous reports by have indicated that veterinary environments and companion animals are notable specimens of *K. pneumoniae*. In our study, 34% of *K. pneumoniae* isolates were detected across different specimens, with a 36% incidence rate of MDR *K. pneumoniae* previously reported by Chaudhry *et al*. [25] in veterinary settings, animal specimens, and cohabiting humans. The occurrence of MDR *K. pneumoniae* in animal specimens supports the notion that the animal gut is a potential reservoir for Enterobacterales, as 33% of fecal samples contained *K. pneumoniae* [34]. Incidence of *bla*NDM-1 harboring *K. pneumoniae* was reported in veterinary specimens, with a 9.4% isolation rate of MDR *K. pneumoniae* from various specimens [34]. Compared with that study, our findings revealed a slightly higher incidence rate of MDR *K. pneumoniae* (14%). This increase could be attributed to the misuse of antimicrobials as growth promoters in food-producing animals, which may inhibit the growth of gut microbiota. In addition, good hygienic practices in veterinary farm settings might have reduced bacterial contamination.

Overall, three different STs were observed in the study, namely, ST29 (7/17; 41%), ST258 (6/17; 35%), and ST11 (4/17; 23%). Previously, we published results consistent with this study, indicating that ST29 is the most prevalent ST in veterinary settings [25]. Similarly, reports from Saudi Arabia identified ST29 as the leading ST of MDR *K. pneumoniae* in Riyadh [35]. The incidence of ESBL-harboring *K. pneumoniae* ST29 in veterinary sources and local meat markets has also been documented in Ghana [36]. In addition, all hvKp isolates in this study had an ST258 allelic profile. The prevalence of ST258 was not surprising, as it is one of the most common STs in *K. pneumoniae* worldwide. Our findings are consistent with those from China, where ST258 was one of the prevalent STs (31%) in veterinary samples [37]. Factors contributing to the global dissemination of ST258 include Type IV pilus, virulence genes, the secretion system, and the restriction-modification system [38].

It was investigated that resistance to various antimicrobial agents, that is, ampicillin, doripenem, cefepime, levofloxacin, ceftriaxone, imipenem, ciprofloxacin, cefotaxime, ceftazidime, gentamicin, amoxicillin, and amikacin, ranged from 35% to 52%, whereas resistance to colistin sulfate was 29%. Although data on resistance patterns to colistin are limited, we found that 11% of the 17 isolates were resistant to colistin, which is considered a last-resort drug for treating *K. pneumoniae* infections. We investigated veterinary samples for the presence of ESBL-producing *K. pneumoniae*. The incidence rate of MDR *K. pneumoniae* in our cohort was consistent with that in a previous study by Schmiedel *et al*. [39]. A previous study by Pulss *et al*. [40] in canines found that carbapenem-resistant *K. pneumoniae* strains detected isolates with acquired resistance determinants, including *bla*OXA-48, *bla*CTX-M-15, and *bla*CTX-M-27.

The limited treatment options for MDR *K. pneumoniae* strains necessitate exploring alternative approaches. This study observed a significant lytic potential of 88% for the PKp-V1 bacteriophage against *K. pneumoniae*. Similar findings were reported in a recent study on carbapenem-resistant MDR *K. pneumoniae*, which identified phages with a broad host range that were effective against these strains [41]. However, studies from Italy and Australia described the vB_Kpn_F48 and AmPh_EK29 phages, which have a narrow host range and are active only against select *K. pneumoniae* strains [42]. TEM analysis revealed that the isolated phage belonged to the Myoviridae family. PKp-V1 demonstrated good stability under varying temperatures, pH values, and chloroform conditions, consistent with a previous study of Kęsik-Szeloch *et al*. [43], which indicated that tailed phages maintain stability across different experimental conditions. In addition, PKp-V1 exhibited notable anti-biofilm activity, showing promising results. These findings are supported by our recent report on KpnM, which also demonstrated anti-biofilm activity against clinical *K. pneumoniae* isolates. These results corroborate with Mulani *et al*. [44] that highlighted the significant anti-biofilm and biofilm disruption efficacy of the PG14 phage against *K. pneumoniae* isolates.

The present study found that the characterized phage had a latent period of 35 min and a burst size of 197 PFU/cell. In contrast, a recent study reported that phages targeting *K. pneumoniae* have a latent period of up to 25 min and a burst size of 80 PFU/cell [45]. The observed differences in burst size between studies may be attributed to variations in phage morphology, as larger bacteriophages typically exhibit lower burst sizes [45].

Although the incidence of ESBL-infected *K. pneumoniae* in veterinary specimens is concerning, the PKp-V1 phage isolated in this study demonstrated promising lytic activity against this pathogen, which has limited treatment options.

## Conclusion

*Klebsiella pneumoniae* is a well-known pathogen that causes significant infections in humans and animals, leading to considerable economic losses worldwide. The high incidence of *K. pneumoniae* in indigenous veterinary specimens poses a pressing health concern for the livestock sector. However, the purified PKp-V1 phage exhibited promising lytic activity against MDR *K. pneumoniae*. Consequently, phage therapy has emerged as a viable alternative treatment option for these infections, given its high specificity toward bacterial species and minimal infectivity toward eukaryotic cells. Overall, our findings underscore the potential of phage therapy as a promising therapeutic approach for managing infections caused by MDR *K. pneumoniae*.

This study faced certain limitations, most notably the absence of whole-genome sequencing (WGS) data for the bacterial isolates due to financial constraints. The lack of WGS data limited our ability to comprehensively understand the genetic diversity and virulence profiles of the *studied K. pneumoniae strains*.

Future research should focus on detailed *in vivo* evaluations to further assess the efficacy and safety of PKp-V1 phage therapy against MDR *K. pneumoniae*. In addition, investigating the potential synergistic effects of phage therapy combined with conventional antibiotics could lead to more effective treatment strategies. These studies will enhance our understanding of the promising role of phage therapy in combating antibiotic-resistant infections and aid in the development of robust therapeutic protocols for the livestock sector.

## Authors’ Contributions

MUT, SM, UAA, MIA, MS, MHR, and BA: Conceptualization and methodology. HE and MK: Data curation and formal analysis and validation. MUT: Methodology and drafted the manuscript. LBE, BBZM, MYME, and HFA: Writing, review, and editing of the manuscript. BA: Supervision. BA, SM, UAA, MIA, MS, and MK: Investigation, MHR and HFA: Project administration. HE, LBE, BBZM, and MYME: Data analysis and revision of the manuscript. All authors have read, reviewed, and approved the final manuscript.
